# Molecular and physiological roles of the adaptor protein CARD9 in immunity

**DOI:** 10.1038/s41419-017-0084-6

**Published:** 2018-01-19

**Authors:** Xiaoming Zhong, Bin Chen, Liang Yang, Zhiwen Yang

**Affiliations:** 1 Jiangxi Province Tumor Hospital, Nanchang, China; 2 Surgery Department, First Affiliated Hospital of Gannan Medical University, Gannan Medical University, Ganzhou, China; 3 Fuzhou Medical College of Nanchang University, Jiangxi, China; 4 Department of Pharmacy, Songjiang Hospital Affiliated Shanghai First People’s Hospital, Shanghai Jiao Tong University, Shanghai, China

## Abstract

CARD9 is a caspase recruitment domain-containing signaling protein that plays a critical role in innate and adaptive immunity. It has been widely demonstrated that CARD9 adaptor allows pattern recognition receptors to induce NF-κB and MAPK activation, which initiates a “downstream” inflammation cytokine cascade and provides effective protection against microbial invasion, especially fungal infection. Here our aim is to update existing paradigms and summarize the most recent findings on the CARD9 signaling pathway, revealing significant mechanistic insights into the pathogenesis of CARD9 deficiency. We also discuss the effect of CARD9 genetic mutations on the in vivo immune response, and highlight clinical advances in non-infection inflammation.

## Facts

CARD9 is a critical adaptor protein that operates downstream of these PRRs in myeloid cells.

CARD9 initiates inflammation cytokine cascade in myeloid cells.

CARD9 exhibits a critical role in human infectious diseases.

## Open questions

What are the underlying mechanisms of CARD9 in microbial infection?

What is the relationship between CARD9 genetic mutations and clinical diseases?

What is the clinical significance of CARD9 in non-infection inflammation diseases?

## Introduction

Innate immune cells are equipped with germ-line-encoded receptors, called pattern recognition receptors (PRRs), which sense the pathogen-associated molecular patterns of foreign pathogens and initiate the host’s immune defenses against invading microbes. Of note, caspase-associated recruitment domain 9 (CARD9) is a critical adaptor protein that operates downstream of these PRRs in myeloid cells. Following receptor engagement, CARD9 selectively interacts with the CARD domain of B-cell CLL/lymphoma 10 (BCL10) and mucosa-associated lymphoid tissue lymphoma translocation protein 1 (MALT1), which triggers an immune response against fungi, bacteria, and viruses^1–5^.

Although considerable progress has been made in determining its functions, CARD9 full characterization remains still unclear. Recently, Roth et al.^6^ confirmed the formation of dsDNA-Rad50-CARD9 signaling complexes in dendritic cells (DCs), revealing a distinct recognition pathway that links DNA sensors to viral infection in a CARD9-dependent manner. Some research groups had found that CARD9 knockdown in neutrophils impaired cytokine release in response to fungal invasion^7,8^. Our group explored the clinical role of CARD9 in aseptic pancreatitis, indicative of its function in non-infectious inflammation^9,10^. Thereby, this review mainly discusses recent advances in determining the role of CARD9 in activating inflammatory reactions.

## CARD9 signaling pathway

### CARD9 as a central adaptor molecule

**Fig. 1:**
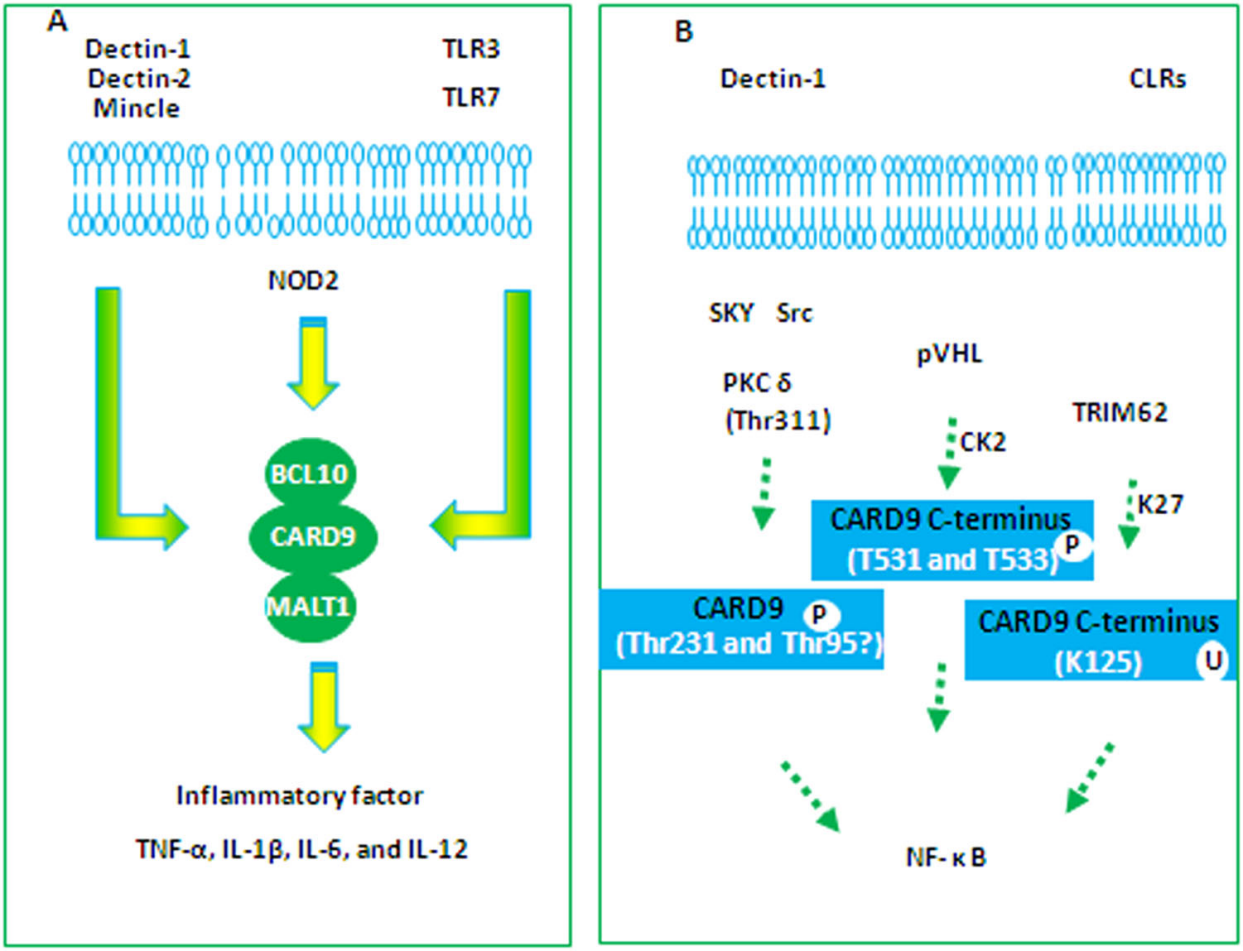
CARD9-mediated signaling in response to microbial infection **a** CLRs/NODs/TLRs-induced CARD9 activation. **b** CARD9 phosphorylation and ubiquitination

There is evidence to suggest that PRR signaling pathways, C-type lectin receptors (CLRs), nucleotide-oligomerization domain (NOD), and toll-like receptors (TLRs), all converge on CARD9 when a microbial infection is encountered (Fig. 1).

CLRs are a class of transmembrane PRRs including Dectin-1, Dectin-2, and Mincle. After CLR recognition, spleen tyrosine kinase (SYK) activates protein kinase C (PKC), the signal-induced assembly of the CARD9/BCL10/MALT1 (CBM) complex. Furthermore, the CBM complex can trigger the activation of NF-κB^1^. Recent studies have indicated that Vav proteins are essential regulators of CLR-induced CARD9-NF-κB in mice and humans. The phosphorylation and activation of Vav proteins may bring CARD9 into the vicinity of key upstream regulators such as PKC and potentially engage CBM complexes for NF-κB control against fungal infection^11^.

Numerous studies have reported that CARD9 is required for NOD2-mediated activation of MAPK signaling components, such as p38 mitogen-activated protein kinase (p38 MAPK) and c-Jun-NH2-terminal kinase (JNK), but not the activation of NF-κB^4^. After treatment with muramyl dipeptide, CARD9-deficient macrophages display normal NF-κB induction but selective defects in p38 and JNK signaling^12^. Thus, CARD9 signaling is involved in the innate immune response against certain intracellular bacteria^4,13^.

TLR3 ligand polyinosinic-polycytidylic acid and TLR7 ligand loxoribine were found to severely inhibit the generation of IL-6, IL-12, and TNF-α in CARD9^−/−^ macrophages, whereas inflammatory cytokines induced by LPS (TLR4 ligand), diacylated lipopeptide FSL-1(TLR2 ligand), flagellin (TLR5 ligand), and CpG DNA (TLR9 ligand) were not defective^3,4^. These results indicated that CARD9 was essential for cytokine secretions through TLR3 and TLR7 but not TLR4, TLR2, TLR5, and TLR9. In addition, the ability of TLRs to recruit CARD9 may be partly explained by other adaptors or enzymes that interact with CARD9 being differentially expressed. In this study, receptor-interacting protein 2 was also found to be essential for CARD9-dependent MAPK activation ‘downstream’ of TLRs^2^. Furthermore, CARD9 signaling was shown to cooperate with TLR/MyD88 signaling to enhance immune cell activation^14^.

### CARD9 proteins are modified by phosphorylation

Protein kinase Cδ (PKCδ), a PKC isoform, has recently been identified as the molecular link between SYK and CARD9 in bone marrow-derived macrophages (BMDMs) from mice^1^. Upon Dectin-1 ligation activation, SYK directly induced PKCδ phosphorylation at position Tyr311. Next, reconstitution experiments using WT-CARD9, CARD9 (T231A), and CARD9 (S303A) mutants demonstrated that PKCδ phosphorylates CARD9 at position Thr231 and Thr303^15^ (Fig. 1). Because global proteomic profiling revealed CARD9 phosphorylation at Thr95 in vivo^16^, it could be speculated that another PKCδ target site could be Thr95. However, due to unsuccessful attempts to express the CARD9 (T95A) mutant in mammalian cells, it was not possible to establish the physiological function of Thr95 phosphorylation.

Casein kinase 2 (CK2)-mediated phosphorylation of CARD9 was also found in renal carcinoma cells. In this study, the VHL tumor suppressor protein (pVHL) was found to serve as an adaptor that promoted the phosphorylation of the CARD9 C-terminus by CK2. pVHL was reported to be a CK2 substrate^17^, bringing CK2 into proximity with CARD9. The CARD9 C-terminus was composed of multiple threonine and serine residues that resembled CK2 phosphorylation sites. CK2 phosphorylated the CARD9 peptide at T531 and T533^18^. Phosphorylation of the CARD9 C-terminus by CK2 was responsible for CARD9-BCL10 complex assembly (Fig. 1).

### CARD9 proteins are modified by ubiquitination

**Fig. 2:**
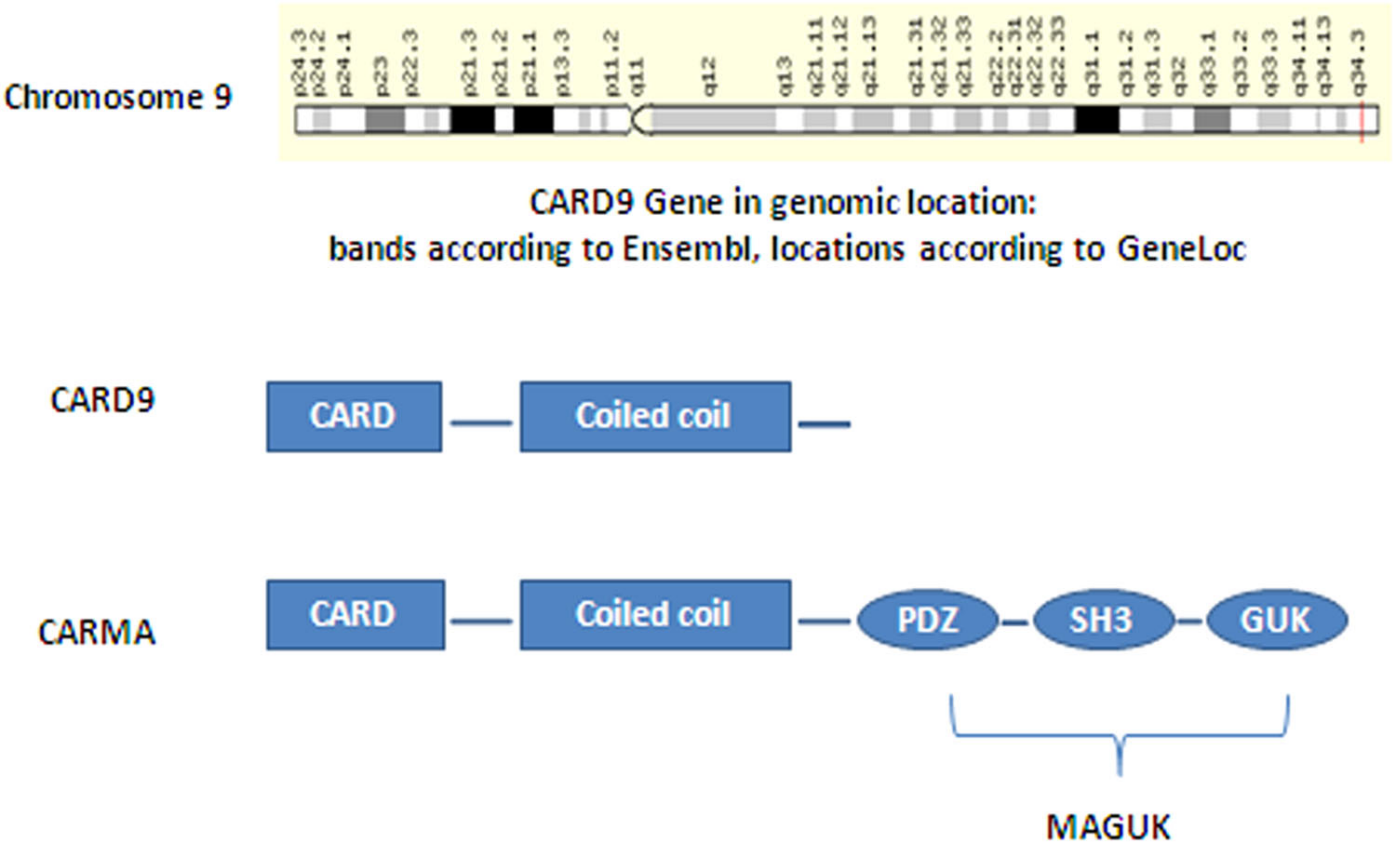
The structure of CARD9 and the genomic location of the CARD9 gene

The N-terminal portion of CARD9 has been identified as very important in the recruitment of BCL10 and MALT1 to form a CBM complex. However, CARD9 has no clear domain within its C terminus composed of an oligomerization domain and its mode of regulation is not fully defined^1,12^ (Fig. 2). A recent study indicated a rare mechanism that involved TRIM62-mediated K27-linked ubiquitination of the CARD9 C-terminus^19^. TRIM62 was identified as a novel binding partner with the CARD9 C-terminus (aa 416–536), and facilitated K27-linked polyubiquitination of CARD9. TRIM62 ubiquitinated CARD9 at K125 and, in turn, a CARD9 mutation at this residue (K125R) abrogated CARD9-induced NF-κB signaling. Furthermore, *Trim62*^−/−^mice showed significant impairment in CLR-CARD9-dependent cytokine production. In parallel, the CARD9 Δ11 variant acted in a dominant-negative manner, disrupting the CARD9-TRIM62 interaction and abolishing TRIM62-mediated ubiquitination (Fig. 1)^19^.

### DsDNA/Rad50/CARD9 signaling complexes for NF-κB activity

The presence of double-stranded DNA (dsDNA) in the cytoplasm, which is normally a DNA-free microenvironment, triggers the production of IL-1β against viral and bacterial invasion. Rad50 is a DNA-binding protein located in the nucleus that is involved in the response to eukaryotic DNA damage.

**Fig. 3:**
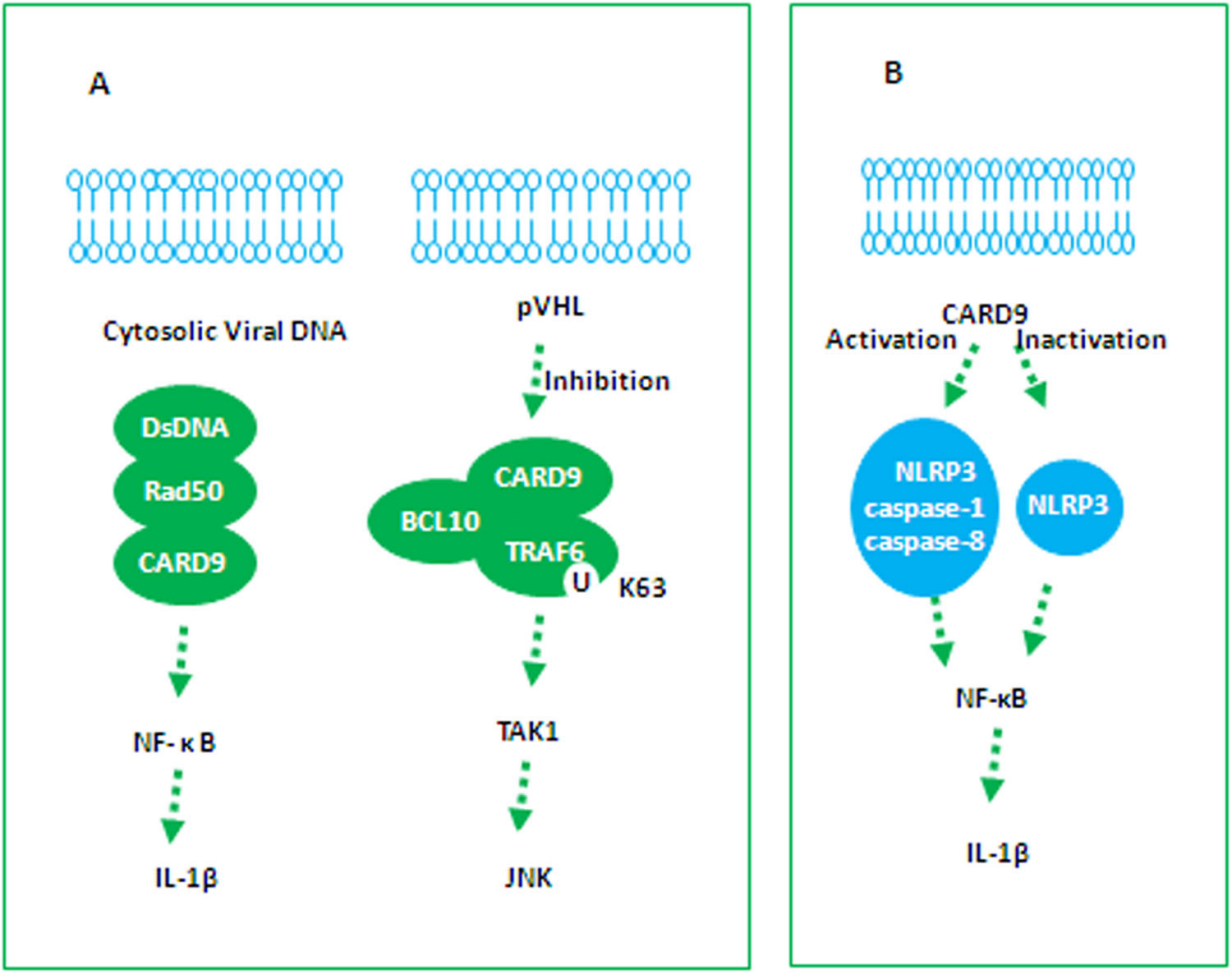
Formation of the CARD9 signaling complex **a** DsDNA/Rad50/CARD9 and TRAF6/BCL10/CARD9 signaling complexes. **b** CARD9-mediated inflammasomes

Roth et al. defined a distinct DNA recognition pathway through the formation of dsDNA-Rad50-CARD9 signaling complexes in DCs^6,20^. After the transfection of DCs with virus DNA, Rad50 translocated rapidly from the nucleus to the cytoplasm, forming a DNA-Rad50 complex. Following the cytosolic localization of Rad50 and DNA, CARD9 was recruited and interacted directly with Rad50 via its Zn-hook region. The formation of viral dsDNA-Rad50-CARD9 complexes mediated NF-κB activation for IL-1β generation. Crucially, Rad50 or CARD9-deficient DCs significantly impaired IL-1β production in response to pathogen DNA. Thus, the Rad50/CARD9 complexes, as viral cytoplasmic DNA sensors, were critical for the host immune response (Fig. 3).

### TRAF6/BCL10/CARD9 signaling complexes for JNK activity

This study showed that CARD9 induced a multiprotein complex with BCL10 proteins, which recruited TRAFs in pVHL-deficient renal cell carcinomas (RCC). After the mitigation of inhibitory phosphorylation of CARD9 by CK2, CARD9-TRAF6 complexes form. Furthermore, CARD9 expression mediated TRAF6 polyubiquitination through K63 linkages. In turn, CARD9 knockdown directly led to an obvious reduction in TRAF6 K63 polyubiquitination in pVHL-deficient cells. As TRAF6 was known to function upstream of TAK1^21^, the formation of a CARD9/BCL10/TRAF6 complex as a proximal signal triggered the sequential activation of TAK1, MKK4, and JNK. This finding indicated that CARD9-dependent TRAF6 drove JNK activity in RCC (Fig. 3)^22^.

### CARD9-mediated inflammasome-induced IL-1β production

CARD9 plays the positive roles in fighting fungal and viral infections through NLRP3 inflammasome. The mycobacterial synthetic adjuvant analog trehalose-6,6′-dibehenate triggers SYK and CARD9 signaling molecules and relies on the C-type lectin Mincle. Trehalose-6,6′-dibehenate could induce NLRP3 inflammasome-dependent IL-1β secretion in BMDMs, in which CARD9 had been identified as an essential mediator^23,24^. *Microsporum canis* (a pathogenic fungus) was found to mediate IL-1β production in the human monocytic cell line THP-1, which was also dependent on the NLRP3 inflammasome. The pathways involved required Dectin-1, SYK, and CARD9, and CARD9 in particular exerted a critical role in the upregulation of pro-IL-1β^25^. *Agrocybe aegerita* lectin isolated from the edible mushroom *A. aegerita* promoted inflammatory cytokine secretion by regulating macrophage activation. *A. aegerita* lectin likely targeted macrophages through Mincle receptor, resulting in SYK/CARD9 signaling activation, which then coupled to NLRP3 and upregulated IL-1β synthesis^26^. Oxidized low-density lipoprotein-mediated IL-1β production also converged on CARD9 (Fig. 3)^27^.

In contrast to the above report, Milton et al. identified a negative regulatory role for CARD9 on IL-1β production. CARD9 markedly down-regulate NLRP3-induced IL-1β in response to *Salmonella* infection in BMDMs. The potential mechanism involved reducing NLRP3 activation via modulation of SYK and Caspase-8 activity^28^.

In addition, other CARD9-independent inflammasomes, Caspase-8 and caspase-1, have been detected. First, Dectin-1 receptors induced the activation of a noncanonical Caspase-8 inflammasome in response to fungal and mycobacterial infection^29^, following the formation of CARD9-BCL-10-MALT1 and the recruitment of MALT1-Caspase-8, in which ASC was required in this scaffold for noncanonical Caspase-8-induced IL-1β^30^. Second, RIG-I served as an RNA virus sensor that could also trigger CARD9 and Caspase-1inflammasome signaling for IL-1β production (Fig. 3)^31^.

### Dectin-1 induces the CARD9-ERK pathway

It established that Dectin-1induces CARD9-ERK activation against fungal infection. CARD9 regulated Dectin-1-mediated ERK activation by linking Ras-GRF1 to H-Ras, which was dispensable for NF-κB activation induced by *curdlan* or *Candida albicans* yeast. Specifically, Dectin-1 initiated SYK-dependent Ras-GRF1 phosphorylation, and then the phosphorylated Ras-GRF1 induced H-Ras recruitment and activation by forming a complex with CARD9, which finally led to ERK phosphorylation^32^. In addition, the Dectin-1-induced SYK-CARD9-ERK pathway was identified during the process of conversion from tumor-associated macrophages into an M1-like phenotype^33^.

### Rubicon-dependent feedback inhibition

The Rubicon protein binds to the Beclin-1complex, and localizes to late endosomes/lysosomesin phagocytic cells. Rubicon serves as a negative regulator of the maturation step of autophagy or the endocytic pathway, depending on the environmental stimulus^34^, and contains a RUN domain, a coiled-coil domain, an N-terminal serine-rich region, a helix-coil-rich region, a C-terminal serine-rich region, and a cysteine-rich region. Yang et al. reported that Rubicon negatively regulated inflammatory cytokine production in response to Rig-I-dependent sensing of RNA viruses and Dectin-1-dependent fungal ligands by displacing CARD9 from the CBM complex, thereby terminating the CARD9-mediated signaling pathway^35,36^. This displacement was dependent on the serine phosphorylation status of Rubicon, which bound to 14-3-3β, known as a phospho-serine/threonine binding protein. Indeed, S_248_-phosphorylation of Rubicon within an N-terminal serine-rich domain completely abolished its interaction with 14-3-3β, which led Rubicon to associate constitutively with CARD9. Furthermore, Rubicon robustly interacted with CARD9 through its C-terminal helix-coil-rich region, disassembling the CARD9-BCL10 signaling complex. Thus, Rubicon acted as a specific feedback inhibitor of CARD9-mediated PRR signal transduction, and markedly suppresses IL-6, IL-1β, and TNF-α production. It remains unclear whether molecular mechanisms specifically alter the binding partner of Rubicon from 14-3-3β to CARD9. It is possible that the interaction of Rubicon with 14-3-3β during the initial period of microbial stimulation, in which Rubicon sterically hinders a CARD9 interaction, and permits CBM signal transduction, triggers NF-κB activation. At the late stage, Rubicon dephosphorylates serine 248, dissociating from the 14-3-3β complex to allow an interaction with CARD9, thereby disrupting CBM signaling. Other unknown proteins and/or alterations in subcellular localization may be attributed to the change of interactions of Rubicon with 14-3-3β and CARD9^35^.

### Troglitazone-dependent negative feedback

The PPAR-γ ligand troglitazone impaired Dectin-1-mediated NF-κB and MAPK activation in human monocyte-derived DCs through the inhibition of CARD9 expression. CARD9 protein expression in the cytosol was dramatically downregulated in troglitazone-treated monocyte-derived DCs. The reduction in CARD9 protein was not due to proteasomal degradation and transcript levels. Thereby, troglitazone, as a negative feedback regulator, helps to alleviate the incoming inflammatory signaling pathways^37^.

### Functional role of CARD9 in neutrophils

Patients with CARD9-deficient polymorphonuclear neutrophils (PMNs) have been found to be highly susceptible to systemic candidiasis that specifically targets the central nervous system (CNS). A novel CARD9 missense mutation (p.R57H; c.170 G > A) was reported in an11-year-old girl who was suffering from *Candida* meningoencephalitis infection in the CNS^7^. In this case, the c.170 G > A CARD9 mutation did not prevent the generation of full-length CARD9 proteins, but imparted CARD9 function. As a result, CARD9 mutations dramatically decreased IL-6, IL-1β, and TNF-α production upon fungal stimulation. Furthermore, CARD9-deficient patients had been found to be significantly impaired in the induction of neutrophil-recruiting CXC chemokines in the cerebrospinal fluid, leading to an absence of neutrophil accumulation in the infected CNS, and the impaired killing of unopsonized *Candida albicans*. A 13-year-old, CARD9-deficient, Asian girl suffering from chronic invasive *Candida* infection of the brain was reported in 2013^38^. Molecular analysis revealed two previously undescribed mutations, c.214 G > A and c.1118 G > C, in CARD9. CARD9-deficient PMNs exhibited selective killing of unopsonized *C. albicans* conidia, which was independent of NAPDH oxidase-derived reactive oxygen species generation.

*Moraxella catarrhalis* as an important causal agent of exacerbations in chronic obstructive pulmonary disease could induce CARD9-dependent NF-κB activation in human granulocytes^39^. CARD9-deficient PMNs isolated from patients exhibited defects in proinflammatory cytokine production and *Phialophora verrucosa* killing^40^. Furthermore, CARD9-deficient patients with spontaneous CNS candidiasis had been successfully treated by granulocyte-macrophage colony-stimulating factor treatment of CARD9-dependent pathways^41^. Finally, Roel et al. demonstrated that human PMNs were primarily responsible for phagolysosomal killing of *C. albicans* through two independent mechanisms, complement receptor 3-mediated uptake of unopsonized *C. albicans* and FcγR-mediated uptake of serum-opsonized *C. albicans*^42^. The first mechanism was found to be dependent on complement receptor 3 via PI3K, SYK, and CARD9, but completely independent of phagocyte NADPH oxidase activity. The second mechanism strictly depended on Fcγ receptors and reactive oxygen species formation by the NADPH oxidase system, in which SYK played a positive role but PI3K does not.

Investigation of mice harboring CARD9-deficient PMNs further confirmed the high susceptibility to invasive fungal infection^7^. Subsequently, research groups provided direct evidence that CARD9 signaling induced CXCL chemokine and initiated neutrophil recruitment in response to fungal infection in CARD9 knockout mice^7,43^. In addition, CARD9^−/−^ neutrophils also played a key role in non-infectious inflammation by suppressing autoantibody-induced arthritis and dermatitis in mice^8,44^.

## CARD9 genetic mutations

Human CARD9 genetic mutations may result in protein structure modifications, lower expression, or function loss. CARD9 genetic mutations are associated with inflammatory responses, a susceptibility to fungal infection^45,46^, Crohn’s disease^47,48^, ulcerative colitis^49,50^, intestinal failure^51^, ankylosing spondylitis^52,53^, as well as the severity of pulmonary tuberculosis^54^.

A novel homozygous R101L mutation in CARD9 was identified in a Brazilian patient with deep dermatophytosis, resulting in impaired fungal killing^55^. Some patients have been reported to harbor a homozygous Q295X point mutation in CARD9, which led to increased susceptibility to dermatophytes^56^ and invasive chronic *Candida*^57^. One particular variant in the CARD9 gene, c.191-192InsTGCT, p.L64fsX59, was associated with *Corynespora cassiicola* infection in a Chinese patient^58^. Patients with the R70W CARD9 mutation were associated with impaired NF-кB activation, which resulted in a defective antifungal response and susceptibility to chronic, invasive fungal infections^59^. The CARD9c.3 G > C mutation predisposed patients to extrapulmonary *Aspergillus* infection in the abdomen and brain, along with reduced neutrophil recruitment to these fungal-infected tissues^60^. Another CARD9 mutation, Ser12Asn, rs4077515, was found to increase susceptibility to idiopathic recurrent vulvovaginal *candidiasis*, a common symptom of *Candida* infection^61^. The CARD9 S12N (c.35 G.A, rs4077515) mutations, an amino acid substitution from a serine to an asparagine residue, was strongly linked to the development of candidemia^46^. A homozygous R18W CARD9 mutation in patients who had functional CARD9 deficiency was identified as a risk factor for invasive *Exophiala* infection^62^.

Recent studies have demonstrated that CARD9 mutations are closely associated with Crohn’s disease and ulcerative colitis development. The genetic mutations c.IVS11 + 1 G > C could cause the loss of function of CARD9, which resulted in diminished immune responses and significant protection against IBD. However, a high-risk variant S12N may induce overexpression of CARD9, which led to a hyper-reactive immune state and high susceptibility to IBD^49,63^. CARD9 mutations (rs10870077 and rs10781499) were significantly associated with both Crohn’s disease and ulcerative colitis^64^, but showed no significant association with IBD in the Chinese Han population^50^. Interestingly, a novel rare non-synonymous variant rs200735402 in CARD9 was shown to play a functionally protective role in Crohn’s disease^47^. CARD9 variant rs4077515 may contribute to ileal Crohn’s disease susceptibility, positive regulation of TNF-α and IL-6 production, and positive regulation of the innate immune response^65^.

The CARD9 mutations, rs4077515, were found to be protective against intestinal failure. One possibility was that this genetic mutations decreased the level of sustained conjugated hyperbilirubinemia and inhibited NOD_2_-dependent signal pathways^51^. The CARD9 mutations rs4077515 significantly decreased the ankylosing spondylitis risk in a HLA-B27-negative Iranian population, possibly by contributing to the CARD9-IL23 axis for the pathogenesis of inflammatory disorders^52^. CARD9 genetic variants, rs4077515, rs10781499, and rs10870077, were found to increase susceptibility to tuberculosis and the severity of pulmonary tuberculosis in a Caucasian cohort of Romanian ethnicity^13,54^.

## Importance of CARD9 in non-infection-related inflammation

Emerging studies indicated that CARD9 was also associated with sterile inflammation disease in the absence of pathogen infections (Table 1).

**Table 1:** CARD9 in sterile inflammation disease

Gene	Disease	Inflammatory factor	Refs.
*CARD9*	Cardiovascular disease	Upregulation	70–72
*CARD9*	Crohn’s disease and ulcerative colitis	Downregulation	73–76
*CARD9*	IgA nephropathy	Upregulation	77
*CARD9*	Autoimmune polyendocrinopathy		
	candidiasis ectodermal dystrophy	—	78
*CARD9*	Autoimmune uveitis	—	79
*CARD9*	Ankylosing spondylitis	—	53
*CARD9*	Primary sclerosing cholangitis	—	80
*CARD9*	Colonic cancer	Upregulation	83
*CARD9*	Gastric mucosa-associated lymphoid		
	tissue lymphomas	Upregulation	84,85
*CARD9*	Kidney tumor	Upregulation	18,22
*CARD9*	Pancreatitis	Upregulation	9,10
*CARD9*	Hypersensitivity	Upregulation	89
*CARD9*	Obesity	Upregulation	90

### Cardiovascular disease

Early cardiovascular pathologies are characterized by inflammatory cell infiltration into the tissues^66^. During this process, the circulating immune cells including neutrophils and monocytes/macrophages follow a cascade of tethering, rolling, arrest, and adhesion, and display a close interaction with endothelial cells in the inflamed tissues^67,68^. Several studies showed that macrophages played a critical role in initiating inflammation in grafted veins. Depletion of macrophages and inhibition of proinflammatory cytokine expression could attenuate neointima formation in rat vein grafts^69^. In this study, CARD9 was highly expressed in the macrophages of grafted veins. Furthermore, necrotic smooth muscle cells induced an inflammatory reaction in macrophages via the CARD9-dependent NF-κB signaling pathway. Finally, CARD9^−/−^ mice significantly inhibited neointima formation of vein grafts by suppressing the migrtion and proliferation of smooth muscle cells. These data suggested that CARD9 mediated necrotic smooth muscle cell-induced inflammation and contributed to neointima formation in vein grafts^70^. Another study showed that angiotensin II systems contributed to all stages of the inflammatory response, leading to cardiac fibrosis, hypertensive cardiac remodeling, and heart failure. Furthermore, CARD9 markedly inhibited the cardiac inflammation and fibrosis induced by angiotensin II infusion. CARD9^−/−^ mice markedly inhibited the expression of collagen I and TGF-β, indicating that CARD9 attenuated cardiac fibrosis by inhibiting myofibroblast formation. CARD9 was also involved in angiotensin II-mediated cardiac inflammation through the regulation of NF-κB and MAPK activity in peritoneal macrophages^71^. Recently, CARD9 was found to suppress obesity-related cardiac hypertrophy through decreasing CBM formation and p38 MAPK production^72^.

### Autoimmune disease

Crohn’s disease and ulcerative colitisare thought to be chronic relapsing immune-mediated diseases arising from dysregulated intestinal immune responses to the gutflora in genetically susceptible genetic backgrounds, which are characterized by inflammation and ulceration of the gut mucosa. Crohn’s disease and ulcerative colitis patients usually show dysregulated microbiomes that may contribute to the dysregulation of the immune system. The CARD9 locus encodes a key pattern recognition receptor of the innate immune system, and specific variants of the *CARD9* gene are involved in Crohn’s disease and ulcerative colitis pathogenesis. CARD9-null mice showed increased vascular leakage and epithelial permeability at the site of injury, delayed intestinal injury recovery, and a defect in epithelial responses due to the impaired expression of IL-22, IL-17A, IL-6, and RegIIIγ. As a result, CARD9 played a pivotal role in intestinal homeostasis, mediating intestinal epithelial cell restitution, innate lymphoid cell and T-helper 17 cell responses, maintenance of the intestinal mucosal barrier, and control of intestinal bacterial infection^73–76^. The gut microbiota in CARD9^−/−^ mice failed to activate the tryptophan metabolites that served as aryl hydrocarbon receptor ligands, which was responsible for the hypersusceptibility of mice to colitis. Patients with crohn’s disease and ulcerative colitis were observed to reduce the production of aryl hydrocarbon receptor ligands and tryptophan metabolites. These results suggested that CARD9 gene disorder altered the composition of the gut microbiota, affected the production of microbial metabolites, and increased sensitivity to colitis^73–76^.

IgA nephropathy is the most common form of primary glomerulonephritis, which is characterized by deposition of IgA-containing immune complexes in the glomerular mesangium. One study reported a genome-wide association study of IgA nephropathy, and follow-up in 20,612 individuals of European and East Asian ancestry. A significant association was detected between the CARD9 gene and the IgA nephropathy risk. A high-risk allele, rs4077515, was discovered, resulting in a p.Ser12Asn substitution in CARD9. This substitution induced the increased expression of CARD9 in monocytes, which were involved in activation of canonical pathways^77^. In addition, the CARD9 gene has also been associated with other immune-related diseases, such as autoimmune polyendocrinopathy candidiasis ectodermal dystrophy^78^, autoimmune uveitis^79^, ankylosing spondylitis^53^, and primary sclerosing cholangitis^80^.

### Cancer

Interestingly, *CARD9* as an inflammation-related gene also promote the proliferation and migration of tumor cells, induce cancer, and affect tumor severity and prognosis^81^.

The APC^min^ (multiple intestinal neoplasia, min) mouse harbors a point mutation in the murine homolog of the adenomatous polyposis coli (APC) gene and is an animal model for studies of human familial adenomatous polyposis, precancerous lesions associated with small-intestinal and colonic cancer^82^. CARD9 had been shown to play a key role in APC^min^ tumor incidence and progression, markedly reducing viability and promoting colonic tumorigenesis in male mice. The positive effect of CARD9 on tumor multiplicity and burden was due to decreased plasma IL6, G-CSF, and RANTES levels, and decreased macrophage and T-cell infiltration into the tumor^83^.

Gastric mucosa-associated lymphoid tissue (MALT) lymphomas frequently occur in translocation-negative tumors. Studies had shown that aberrant CARD9 upregulation may contribute to the development or progression of MALT lymphomas mediated by activation of the NF-isB signaling pathway, especially among patients who were not infected with *Helicobacter pylori*^84,85^. RCC is a common form of kidney tumor. Several lines of evidence suggested that CARD9 linked the VHL tumor suppressor protein (pVHL) to trigger tumor-related signaling molecule, thereby controlling RCC cell growth. pVHL, which was expressed upstream or parallel to CARD9, and was bound to CK2, promoted the phosphorylation of CARD9. Consequently, VHL-deficient cancer cells failed to phosphorylate CARD9, resulting in constitutive activation of CARD9, an upstream trigger for NF-κB activation to induce tumor occurrence^18^. Another study reported that pVHL^+/+^ RCCs sequentially stimulate CARD9 and TRAF and the JNK signaling pathway in a hypoxia-inducible factor alpha (HIFα)-independent manner. In the context of pVHL loss, CARD9 silencing led to a sharp reduction in TRAF6, which was known to function upstream of JNK, retarding tumor growth^22^.

Macrophages are critical immune effector cells that respond to the tumor microenvironment. Substantial experimental and clinical evidence indicates that tumor cell metastasisis associated with the presence of a high number of macrophages in various cancers and dynamic changes in the specific phenotypes of macrophage subpopulations^86^. High expression of CARD9 was reported in tumor-infiltrating macrophages and clinicopathologic analysis of colon cancer patients suggested that CARD9 expression was strongly correlated with tumor progression. Furthermore, CARD9-null mice provided evidence that CARD9 facilitated liver metastasis of colon carcinoma cells. Mechanistic studies revealed that CARD9 contributes to tumor metastasis through enhancing metastasis-associated macrophage polarization, independent of the number of infiltrating macrophages. CARD9 polarized macrophages toward a M2 phenotype in the tumor microenvironment by NF-κB activation^87^.

### Pancreatitis

Severe acute pancreatitis (SAP) is characterized by a progressive inflammatory response with a high mortality rate^88^. To the best of our knowledge, the early stages of SAP manifest a sterile inflammation in response to endogenous substances. It is currently accepted that the activation of mononuclear cells is an early event in SAP patients, which induces an inflammatory cascade that leads to systemic organ failure. In our studies, mononuclear cells isolated from SAP patients were found to overexpress CARD9 that may serve as an upstream molecule of NF-κB and p38. What was more, CARD9 overexpression was positively correlated with the outcome and severity of pancreatic injury in SAP patients^10^. In another study, siRNA silencing of the CARD9 gene in SAP rats was used to investigate the therapeutic effects and potential mechanisms of CARD9. Interestingly, our findings provided strong evidence that blocking CARD9 expression failed to trigger NF-κB and p38 activity, which could effectively alleviate pancreatitis severity, as well as liver and lung injury^9^.

### Hypersensitivity

Allergic contact dermatitis is caused by the reaction of T cells to various allergens. Allergens can effectively penetrate the skin and bind covalently to skin proteins to form hapten. Skin-resident DCs can recognize haptenated proteins, transfer to the skin-draining lymph nodes, and then prime hapten-specific T cells. This process is called sensitization, and indicates that DCs are essential for allergic contact sensitization to haptens. However, the mechanism by which haptens stimulate DCs to sensitize T cells remains unclear. In this study, CARD9 overexpression in DCs increased their ability to prime T cells. The potential signaling pathway was found to be the coupling of ITAM-SYK-CARD9 signaling to IL-1 secretion in DCs, involving the sequential activation of ITAM, SYK, CARD9, BCL10, the NLRP3 inflammasome, and IL-1 signaling. DC-specific deletion of CARD9 was sufficient to abolish hapten-induced IL-1 secretion and hinder allergic contact sensitization to haptens^89^.

### Obesity

Obese patients are associated with low grade chronic inflammation, known as “metabolic inflammation”, the heightened infiltration of macrophages, which has detrimental effects on metabolism and cardiovascular dysfunction. A recent study reported that CARD9^−/−^ mice had significantly decreased numbers of infiltrating macrophages in the heart, which prevented myocardial dysfunction and ameliorated high fat diet-induced insulin resistance and glucose intolerance, leading to alleviation of high fat diet-induced obesity potentially through CARD9-dependent p38 suppression^90^.

## Conclusion

In summary, evidence is emerging of the molecular and cellular mechanisms of CARD9 activation of inflammatory reactions, particularly regarding cytosolic DNA-recognition signaling, the ubiquitination pathway, negative feedback regulation, genetic mutations, and aseptic inflammation. However, important questions still remain, such as (1) how CARD9 is translocated from the cytoplasm to the nucleus, (2) the role of CARD9 in the early and late inflammatory phases, and (3) the nature and mechanism by which CARD9 is involved in non-infection diseases, such as tumor development and cardiac fibrosis.
