# Author Correction: Molecular and physiological roles of the adaptor protein CARD9 in immunity

**DOI:** 10.1038/s41419-019-1401-z

**Published:** 2019-02-18

**Authors:** Xiaoming Zhong, Bin Chen, Liang Yang, Zhiwen Yang

**Affiliations:** 1 Department of Pharmacy, Songjiang Hospital Affiliated Shanghai First People’s Hospital, Shanghai Jiao Tong University, Shanghai, China; 2 Jiangxi Province Tumor Hospital, Nanchang, China; 3 Surgery Department, First Affiliated Hospital of Gannan Medical University, Gannan Medical University, Ganzhou, China; 4 Fuzhou Medical College of Nanchang University, Jiangxi, China

**Correction to:** **Cell Death Dis.** 9: 52

10.1038/s41419-017-0084-6

published online 19 January 2018

The original version of this Article contained errors in the author affiliations.

Dr. Xiaoming Zhong, Dr. Bin Chen, and Dr. Liang Yang were incorrectly associated with the Department of Pharmacy, Songjiang Hospital Affiliated Shanghai First People’s Hospital, Shanghai Jiao Tong University, Shanghai, China.

This has now been corrected in both the PDF and HTML versions of the Article.

